# Parotid Tuberculosis as the Presenting Manifestation of Disseminated Disease in an Otherwise Asymptomatic HIV Patient

**DOI:** 10.7759/cureus.22493

**Published:** 2022-02-22

**Authors:** Mohammed Ajmal, Shridhar Pattar, Aditya Sudan, Minakshi Dhar

**Affiliations:** 1 Internal Medicine, All India Institute of Medical Sciences, Rishikesh, IND

**Keywords:** lymphadenopathy, parotid mass, disseminated tuberculosis, hiv, parotid tuberculosis

## Abstract

Parotid tuberculosis is not widely described in the literature. Even though a rare presentation of a commonly occurring disease, it is still missed as a differential diagnosis and often mistaken for neoplasms, eventually leading to unnecessary surgical resections. Here, we present a case of a young lady with a slowly growing unilateral parotid mass along with matted, non-tender cervical, axillary, and inguinal lymphadenopathy, who was incidentally found to have HIV with a cluster of differentiation 4 (CD4) lymphocyte count of 38 per microliter. Fine needle aspiration revealed acid-fast bacilli and CT thorax showed features of pulmonary tuberculosis, thus suggesting a disseminated tuberculosis infection. She was started on anti-tubercular and anti-retroviral therapy, after which there was a symptomatic improvement.

## Introduction

Parotid tuberculosis is a rare clinical entity even in developing countries where tuberculosis is endemic and has only been described in the literature as case reports. It often poses a diagnostic challenge, as it is difficult to differentiate from a slow-growing malignancy of the parotid gland. Most diagnoses are made from post parotidectomy histopathologic specimens [1]. Swelling of the parotid glands is common in patients infected with human immunodeficiency virus (HIV) and can occur due to a broad spectrum of conditions, including inflammatory, infective, and neoplastic etiologies. It can even be the initial manifestation of the viral infection itself. The usual presentation involves swelling of the parotid glands, xerostomia, and other constitutional symptoms. However, more often these are due to benign conditions like benign lymphoepithelial cysts (BLEC) [2]. It becomes imperative to clinically identify parotid tuberculosis in the setting of parotid swelling, as the response to medical therapy is excellent and surgical management is rarely required. Here, we report a case of parotid tuberculosis as an initial presentation of HIV.

## Case presentation

A young lady in her mid-thirties with a history of multiple sexual partners presented with a right-sided parotid and neck swelling for three years. Initially, the swelling was small and gradually increased in size over three years. There was no dryness of the mouth. She did not have significant involuntary weight loss, drenching night sweats, fever, cough, or contact with any patient with tuberculosis. She had previously consulted a surgeon for these complaints who advised for fine needle aspiration cytology (FNAC), but she did not follow up for the same. Now she presented with a further increase in the size of the swelling associated with mild discomfort. Examination revealed non-tender, firm, diffuse enlargement of the right parotid gland. The overlying skin was normal. There was no facial nerve weakness. She had ipsilateral enlarged, firm, non-tender, matted lymph nodes in the posterior triangle of the neck. There was no sinus or fistula. She also had right axillary and inguinal lymphadenopathy. The rest of the examination was unremarkable.

**Figure 1:**
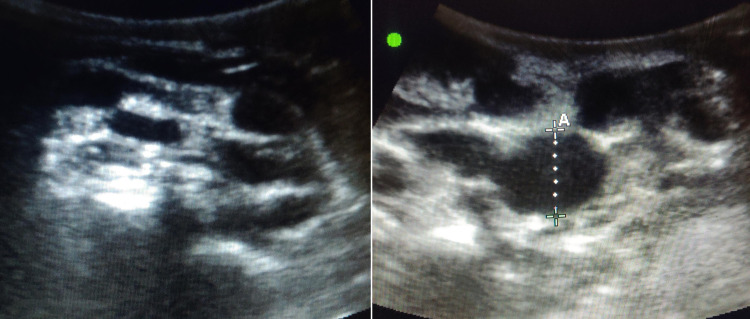
Ultrasound of the parotid showing multiple hypoechoic cystic areas.

Her complete blood counts and liver and kidney function tests, including serum electrolytes, were all within normal limits. Her viral serology was reactive to HIV, and her cluster of differentiation 4 (CD4) lymphocyte count was 38 cells per microliter. Ultrasound of the parotid showed multiple hypoechoic cystic areas with internal echoes (Figure 1).

**Figure 2:**
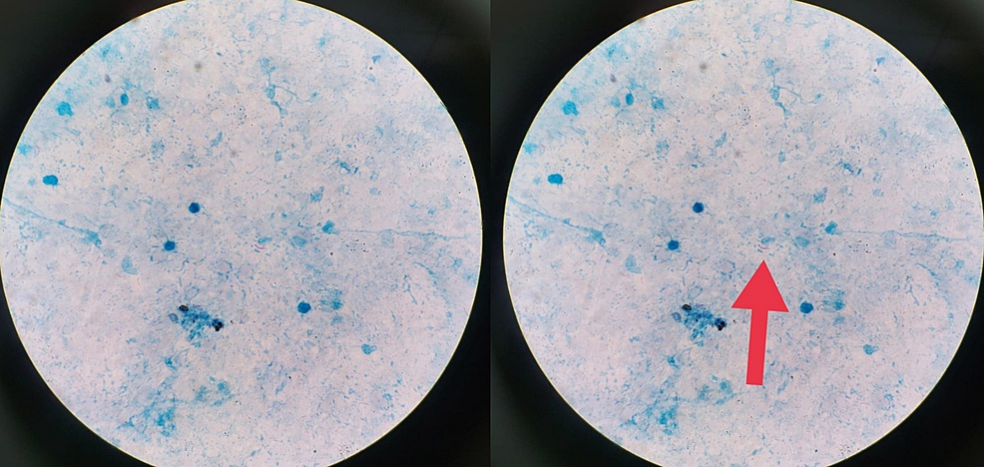
Fine needle aspiration smear showing acid-fast bacilli on Ziehl-Neelsen staining.

FNAC from the right cervical lymph node showed necrotizing lymphadenitis. We went ahead with FNAC of the right parotid gland to rule out other infective and neoplastic etiologies. The smears showed predominant necrosis along with acid-fast bacilli on staining with Ziehl-Neelsen (ZN) stain (Figure 2).

**Figure 3:**
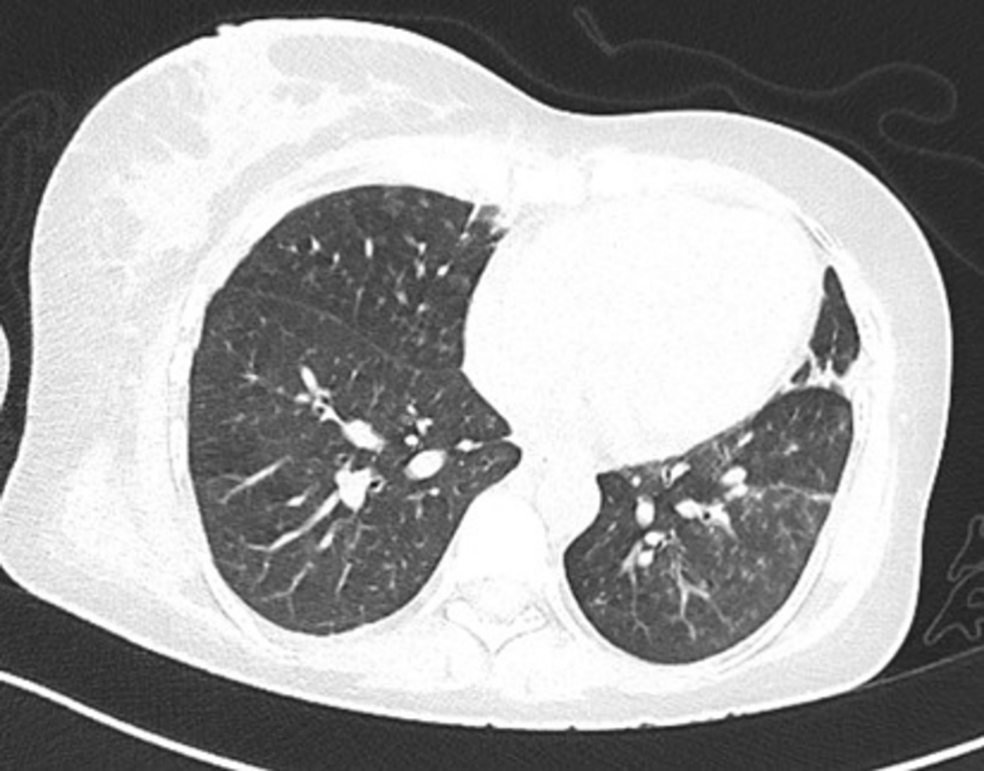
CT thorax showing a tree-in-bud appearance.

A contrast-enhanced computed tomography (CT) (with high-resolution cuts) of the thorax showed multiple heterogeneously enlarged mediastinal lymph nodes and multiple centrilobular nodules giving a tree-in-bud appearance, and few patches of consolidation in the left lower lobe (Figure 3).

Since she did not have any respiratory complaints, we attempted sputum induction, which detected *Mycobacterium tuberculosis* in nucleic acid amplification test from the sputum sample.

Considering the duration of symptoms with the background of an HIV infection, the differential diagnoses kept were HIV-associated parotid swellings including BLEC, diffuse infiltrative lymphocytosis syndrome (DILS), Castleman disease, slow-growing neoplasms of the parotid gland including pleomorphic adenoma, non-Hodgkin's lymphoma, and cystadenocarcinoma, chronic infectious causes including tuberculosis and histoplasmosis, and granulomatous disorders like sarcoidosis. Since our patient had unilateral swelling of the parotid without xerostomia, the possibility of an infection or malignancy was more likely. Though parotid swellings are common in patients with HIV, the presence of matted lymph nodes and the absence of characteristic ultrasound findings prompted a further workup. The need for tissue evidence was emphasized to differentiate between our differential diagnoses. As the smears showed acid-fast bacilli, the diagnosis of a tubercular infection of the parotid gland was confirmed. Her respiratory system examination and chest radiograph were normal. Even though tubercular infection of lymph nodes is common in HIV patients, we decided to screen for a pulmonary focus of infection, the most common site of dissemination. As the CT findings correlated with the clinical scenario, along with the detection of *Mycobacterium tuberculosis* in the sputum, we had finally arrived at the diagnosis of disseminated tuberculosis in our patient.

She was started on anti-tubercular therapy; rifampicin (450 mg), isoniazid (225 mg), pyrazinamide (1200 mg), and ethambutol (825 mg) based on her weight along with pyridoxine supplementation. On the third week after starting anti-tubercular therapy, she was started on tenofovir disoproxil fumarate (300 mg), lamivudine (300 mg), and dolutegravir (50 mg) as part of anti-retroviral therapy. She was followed up on an outpatient basis where there was a reduction in the size of the swelling and regular monitoring of serum levels of liver enzymes.

## Discussion

Salivary gland enlargement could usually be due to infectious, inflammatory, or neoplastic etiologies. Acute painful parotid swelling is commonly seen in viral or bacterial parotitis. On the other hand, gradual painless enlargement typically leads to a suspicion of any neoplastic etiology [3,4]. Nonetheless, one has to keep in mind certain common infections, which may present in this manner, particularly HIV and tuberculosis. This is a special case where parotid tuberculosis was the initial presenting feature of HIV.

Salivary gland involvement is seen in up to 10% of patients with HIV [5]. It usually manifests as painless bilateral or unilateral (as in our patient) salivary enlargement with xerostomia. The causes could be bacterial sialadenitis, BLEC, intra-parotid lymphadenopathy, primary or metastatic non-Hodgkin's lymphoma, Kaposi's sarcoma, adenoid cystic carcinoma, or as a part of DILS, with characteristic infiltration of salivary glands and the lungs with CD8+ T lymphocytes [6]. Unlike other salivary glands, the parotid gland contains lymphoid tissue within the capsule. HIV can cause hyperplasia of the lymphoid tissue leading to parotid enlargement [7]. However, it is essential to differentiate between these lesions, so FNAC plays an important role.

Our patient presented with painless right parotid enlargement, and FNAC was done to rule out neoplasms and infectious causes apart from HIV. However, it revealed acid-fast bacilli. Though tuberculosis can affect most organs in the body, parotid tuberculosis as part of extrapulmonary tuberculosis is quite rare. The first case of parotid gland tuberculosis was reported in 1893 [8]. Since then, only a handful of such cases have been reported. It presents as gradual parotid enlargement, sometimes painful, usually associated with dry mouth, dental caries, difficulty in opening the mouth, and fever. It is often felt as a firm, non-fluctuating, mobile swelling with ill-defined borders [9,10].

Ultrasound of parotid can be used as an initial radiological tool to assess parotid swelling in general and to differentiate between the two types of parotid tuberculosis: parenchymal and periparotid types [10]. The former appears to be diffusely enlarged, hypoechoic gland, sometimes with anechoic zones, with or without cavities. The periparotid variant appears as a hyperechoic parotid gland with hypoechoic nodules located in its peripheral zone [11]. It shows linearly arranged enhancing nodules in the superficial lobes of the glands on CT, and MRI may demonstrate hypointense lesions on T1 and hyperintense lesions on T2-weighted images with homogeneous contrast enhancement [7]. Though these radiological findings are sensitive, they do not differentiate them from neoplasms. Hence, tissue diagnosis is of utmost importance in diagnosing parotid tuberculosis. Ultrasound-guided FNAC is recommended as the initial modality as it has 81-100% sensitivity and 94-100% specificity. It helps establish a diagnosis in most cases as it did in our patient. However, if it fails, an excision biopsy is required [12]. Since it mimics neoplasms, differentiating it from parotid tumors and its early diagnosis is very crucial as the treatment options offered are different. Medical management with anti-tubercular therapy for six months is effective, and surgical resection is seldom required as opposed to parotid neoplasms [13]. However, if FNAC is inconclusive, an excision would be warranted in such cases [10].

## Conclusions

Parotid enlargement is commonly seen in patients with HIV and is often benign. Nevertheless, the treating physician has to rule out the possibility of chronic infections, including tuberculosis, along with malignancies. Even though parotid tuberculosis as an extrapulmonary manifestation is rare, its early recognition is pivotal as it avoids unnecessary surgery, and these patients respond well to anti-tubercular therapy. In the case of extrapulmonary manifestations of tuberculosis, it is encouraged to look for a pulmonary focus even in an otherwise asymptomatic patient so that proper management and monitoring for complications can be planned. CD4 count of the patient was 38 per microliter, and more cases need to be reported to find out if there is any correlation between low CD4 count (less than 50) and parotid tuberculosis in patients with HIV. Routine screening for HIV needs to be emphasized as many patients remain asymptomatic for the disease and present late during the course of illness.
